# Supplementary material for: Robust estimation of 1/f activity improves oscillatory burst detection
Source: Eur J Neurosci. 2022 Oct 11;56(10):5836–52. doi: 10.1111/ejn.15829 (PMC9828710; doi:10.1111/ejn.15829)
Supplement: Supplementary file 1 — Figure S1 Simulations were performed using data with either a linear (A‐C) or non‐linear background 1/f power spectrum. These data were then combined with simulated bursts in the: theta‐band (4 Hz) alone; alpha‐band (10 Hz) alone; or both the theta and alpha‐bands (4 Hz and 10 Hz). The SNR of the bursts was varied between 24 and 48. For each set of simulations, the root‐mean‐squared error (RMSE) between the estimated and actual 1/f fit was plotted for BOSC, eBOSC and fBOSC. Individual data points correspond to RMSE values from each simulated trial. Error bars correspond to standard error. The pattern of results is very similar to those shown in main Figure 2 (where the SNR of bursts varied between 5 and 12). Figure S2 Simulations were performed using data with a non‐linear 1/f power spectrum and embedded oscillations in the theta‐, alpha‐ or theta‐ and alpha‐bands. The error between the estimated and actual 1/f fit was quantified using the Akaike information criterion (AIC) for each method (BOSC, eBOSC and fBOSC). For model comparison, AIC values were compared between fBOSC and BOSC (A); fBOSC versus eBOSC (B); and between BOSC and eBOSC (C). Error bars indicate standard error for each method, and individual datapoints correspond to each simulated trial. Note that fBOSC has lower AIC values than the other two methods, indicating better accuracy at recovering the non‐linear background 1/f activity. Figure S3 An example theta burst detected by fBOSC but not eBOSC. A non‐linear 1/f spectrum was simulated (A) and combined with a simulated burst at 4 Hz (SNR = 9, compared with the background spectrum at 4 Hz) from ~ 8 s to 9 s in this dataset (B). Burst detection was performed using fBOSC and eBOSC using the same parameters as specified in the main manuscript. Detected burst times are plotted in red. fBOSC detected this burst (C) but eBOSC did not (D). Figure S4 Simulations were performed using data with a non‐linear 1/f power spectrum and embedded theta or alpha bursts [file EJN-56-5836-s001.pdf]

Supporting Information

Robust Estimation of 1/f Activity Improves Oscillatory Burst Detection

Robert A. Seymour, Nicholas Alexander, Eleanor A. Maguire

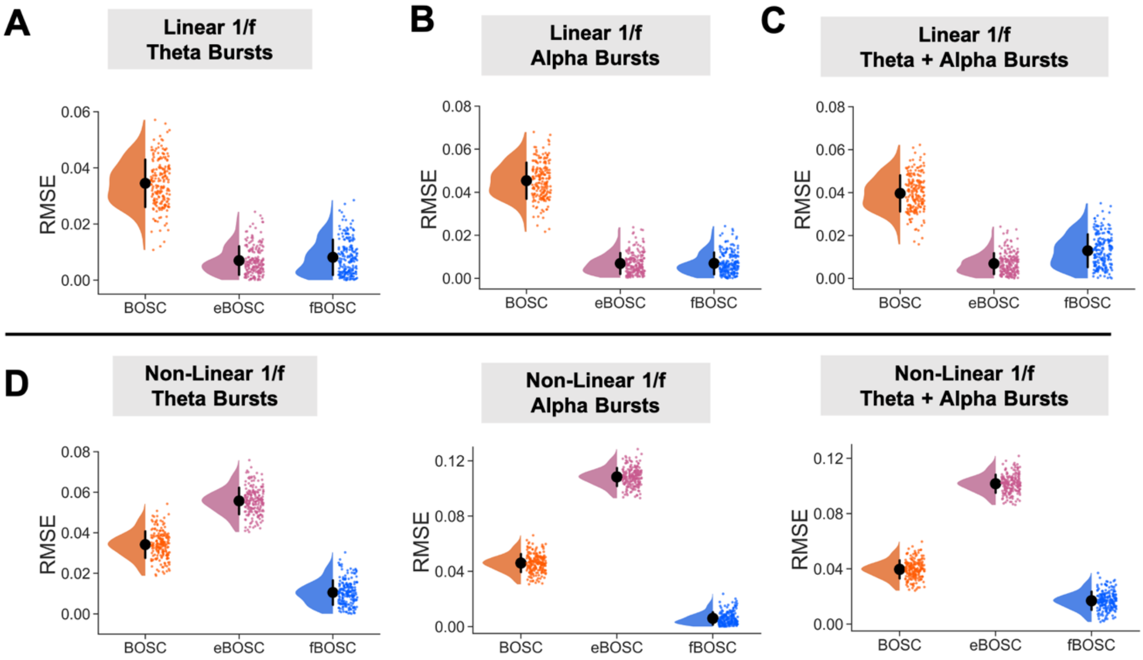

SUPPORTING FIGURE S1 Simulations were performed using data with either a linear (A-C) or non-linear background 1/f power spectrum. These data were then combined with simulated bursts in the: theta-band (4 Hz) alone; alpha-band (10 Hz) alone; or both the theta and alpha-bands (4 Hz and 10 Hz). The SNR of the bursts was varied between 24-48. For each set of simulations, the root mean squared error (RMSE) between the estimated and actual 1/f fit was plotted for BOSC, eBOSC and fBOSC. Individual data points correspond to RMSE values from each simulated trial. Error bars correspond to standard error. The pattern of results is very similar to those shown in main Figure 2 (where the SNR of bursts varied between 5-12).

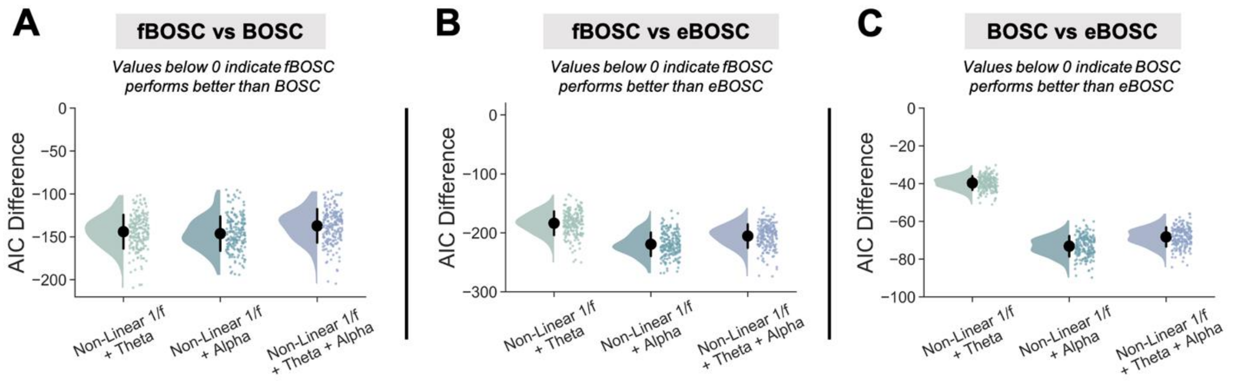

SUPPORTING FIGURE S2 Simulations were performed using data with a non-linear 1/f power spectrum and embedded oscillations in the theta-, alpha- or theta- and alpha-bands. The error between the estimated and actual 1/f fit was quantified using the Akaike information criterion (AIC) for each method (BOSC, eBOSC and fBOSC). For model comparison, AIC values were compared between fBOSC and BOSC (A); fBOSC versus eBOSC (B); and between BOSC and eBOSC (C). Error bars indicate standard error for each method, and individual datapoints correspond to each simulated trial. Note that fBOSC has lower AIC values than the other two methods, indicating better accuracy at recovering the non-linear background 1/f activity.

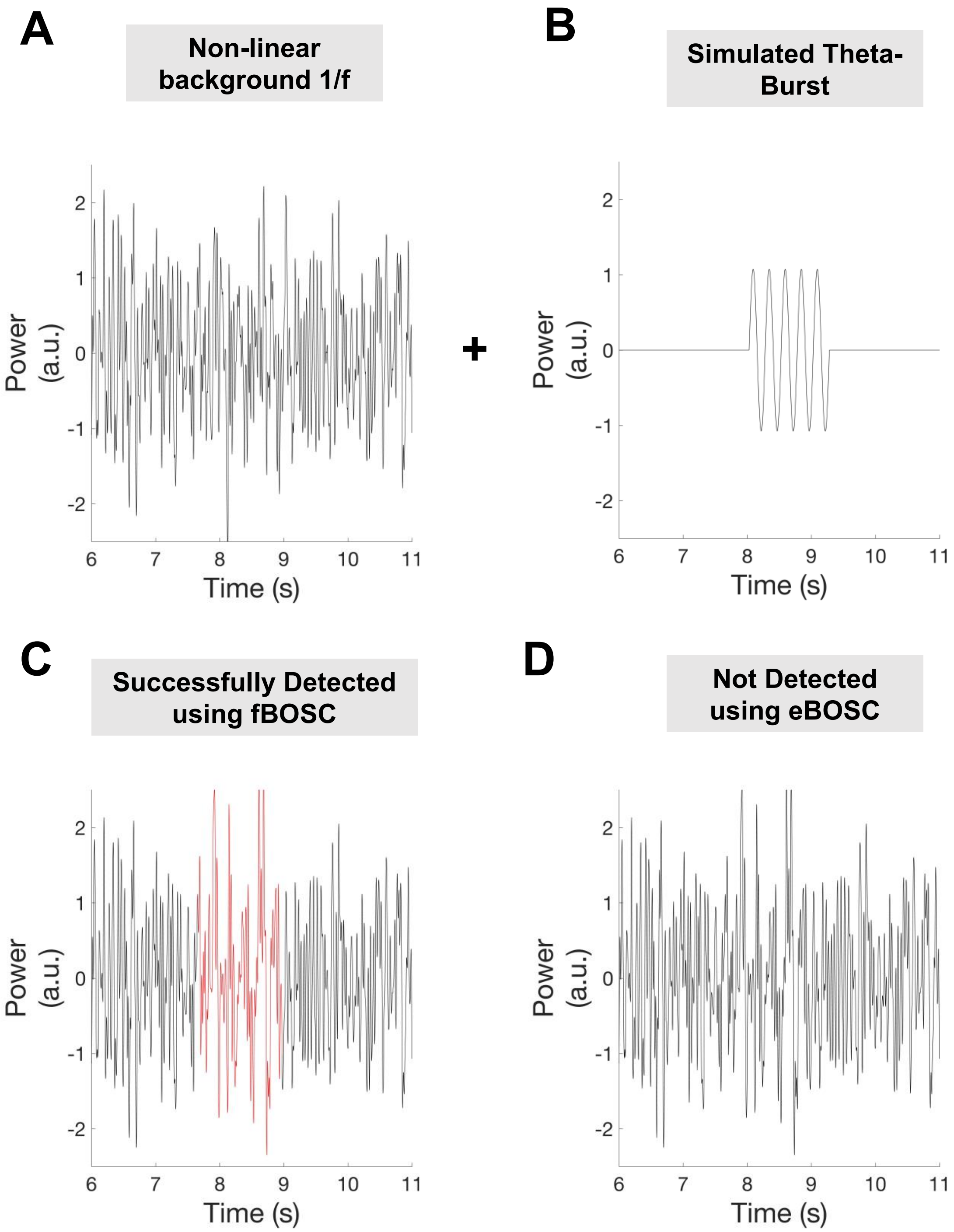

SUPPORTING FIGURE S3 An example theta burst detected by fBOSC but not eBOSC. A non-linear 1/f spectrum was simulated (A) and combined with a simulated burst at 4 Hz (SNR = 9, compared with the background spectrum at 4 Hz) from ~ 8 s to 9 s in this dataset (B). Burst detection was performed using fBOSC and eBOSC using the same parameters as specified in the main manuscript. Detected burst times are plotted in red. fBOSC detected this burst (C) but eBOSC did not (D).

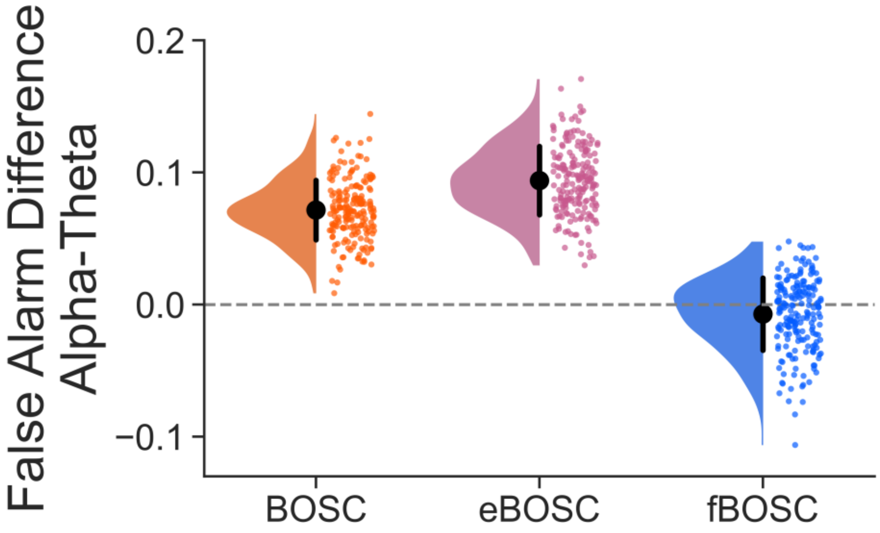

SUPPORTING FIGURE S4 Simulations were performed using data with a non-linear 1/f power spectrum and embedded theta or alpha bursts (the SNR of bursts varied between 24-48). BOSC, eBOSC and fBOSC were used to detect these oscillatory bursts. The difference in false alarm rate between theta and alpha burst detection is plotted, with an additional dotted line at 0. Across all plots, individual data points correspond to each simulated trial. Error bars correspond to standard error. Note that fBOSC is much closer to 0 than the other two methods, indicating smaller differences in the false alarm rate between frequency bands. Hit rate is not plotted because the high SNR bursts caused ceiling effects, i.e. the hit rate was nearly always equal to 1 for each trial.
